# MFDA-UNet: Medical Image Segmentation with Frequency-Decoupled Representation and Gated Cross-Scale Integration

**DOI:** 10.3390/s26103183

**Published:** 2026-05-18

**Authors:** Weiming Deng, Cong Wu

**Affiliations:** School of Science, Hubei University of Technology, Wuhan 430068, China; 102312350@hbut.edu.cn

**Keywords:** medical image segmentation, linear attention, state space models

## Abstract

Convolutional Neural Networks (CNNs) excel at extracting local features, but due to their restricted receptive fields, they often struggle to capture large-scale global context. Transformers leverage self-attention mechanisms to facilitate global interactions, yet the computational cost of standard self-attention scales quadratically with image resolution. To overcome these limitations, we propose MFDA-UNet, which adopts a hybrid architecture of convolution and linear attention for synergistic feature processing. To fully leverage their respective strengths, we design the Mamba-inspired Frequency-Decoupled Attention (MFDA) block. Through frequency decoupling, this block utilizes convolutions to process high-frequency local information, while employing linear attention to model the long-range dependencies of low-frequency global information. To enhance the feature representation capability of linear attention, we construct the Mamba-Enhanced Linear Attention (MELA) block. Inspired by MILA, this block injects Positional Encoding to substitute the forget gate functionality of Mamba and integrates the Mamba block structure into the linear attention mechanism. This design effectively strengthens representational power, accomplishing long-range dependency modeling with highly efficient linear complexity. Furthermore, we introduce the Gated Cross-Scale Attention (GCSA) module to optimize traditional skip connections. It aggregates features via cross-scale linear attention and incorporates Mamba’s high-performance gating mechanism for adaptive feature filtering, achieving precise feature fusion and selection. We conducted extensive experiments on four multi-modal benchmarks: ISIC 2017, ISIC 2018, Synapse, and ACDC. MFDA-UNet achieved improvements in the DSC by 0.44%, 0.15%, 0.53%, and 0.84% across the respective datasets compared to the second-best models. By capturing local and global multi-scale semantics with relatively low computational overhead, MFDA-UNet provides an efficient and robust solution for medical image segmentation.

## 1. Introduction

As medical image segmentation evolves, models based on diverse paradigms continue to emerge. Among these, Convolutional Neural Networks (CNNs), exemplified by U-Net [1], are highly efficient at extracting hierarchical image features. However, the inherent geometric constraints of convolutional kernels limit their ability to model long-range dependencies. Transformers process image features as token sequences, inherently endowing them with robust global contextual awareness. Yet, the computational complexity of standard self-attention scales quadratically with spatial resolution. This creates severe memory and efficiency bottlenecks when processing high-resolution scans. To address this, linear attention mechanisms were developed to reduce complexity from $O(N^{2})$ to O(N) via kernel-based feature mapping. Despite its computational efficiency, previous studies [2,3] have demonstrated that linear attention suffers from limited expressive capacity.

Recently, State Space Models (SSMs) [4] have demonstrated exceptional long-range dependency modeling with linear computational complexity. Mamba, an advanced SSM featuring a selective scanning mechanism, has even outperformed Transformers in various linguistic tasks. Following this, Vision Mamba adapted this architecture for visual tasks with promising results. Recent studies [5,6] suggest that Mamba’s macro-architectural designs, such as its weighted bypass structures, significantly enhance feature extraction in image processing. However, the study also points out that a structural conflict remains within the model: unlike text, visual data lacks a strict unidirectional sequence. Consequently, applying state space models (SSMs) to non-causal 2D tasks such as image segmentation creates a fundamental structural mismatch. In contrast, Transformers can symmetrically compute global interactions across all tokens via their self-attention mechanism, making them inherently more flexible in visual contexts.

Research by Chen et al. [5] reveals a high degree of mathematical equivalence between Mamba and linear attention. Motivated by this insight, we propose the Mamba-inspired Frequency-Decoupled Attention (MFDA) block to address the aforementioned limitations. This block retains Mamba’s macro-block architecture but replaces the core SSM with linear attention. This substitution effectively eliminates the directional constraints of autoregressive models, fully adapting the structure for 2D spatial processing. To maximize the long-range modeling capabilities of linear attention while compensating for its lack of detail extraction, the block decouples input features in the frequency domain. High-frequency components, which contain local edge details, are processed using convolutions. Conversely, the low-frequency global pathway incorporates Conditional Positional Encoding (CPE). This design is inspired by [5], which uses Positional Encoding to replace Mamba’s forget gate. Subsequently, the pathway employs Mamba-enhanced linear attention to model comprehensive long-range dependencies.

From another research perspective, this paper investigates how to effectively adapt Mamba’s gating design into a cross-scale attention mechanism within a U-shaped architecture. The typical encoder–decoder framework naturally generates multi-level hierarchical features. Decoder features encapsulate rich global semantics, whereas encoder features retain high-frequency local spatial details. Based on this characteristic, we propose the Gated Cross-Scale Attention (GCSA) module, leveraging both the efficiency of linear attention and the selective capability of Mamba’s gating mechanism. GCSA employs a cross-scale linear attention mechanism to fuse features from the encoder and decoder, bridging their semantic gap. Subsequently, a gating pathway generated from the decoder features is applied to adaptively filter the fused representations. This ensures precise information aggregation and improves the model’s segmentation performance. The primary contributions of this paper are summarized as follows:

- We propose the Mamba-inspired Frequency-Decoupled Attention (MFDA) block, which performs highly efficient high-low frequency decomposition via a simple average pooling operation. Low-frequency components are augmented with CPE and routed through Mamba-enhanced Linear Attention (MELA) for global context modeling. High-frequency components are processed via a parallel pathway using depth-wise convolutions for local feature enhancement. This dual-path design effectively synergizes the strengths of CNNs and Transformers, achieving a balance between macroscopic structure modeling and fine edge detail perception.
- We design a Mamba-style Gated Cross-Scale Attention (GCSA) module. This module employs a cross-scale linear attention mechanism to efficiently fuse high-level semantics from the decoder with structural details from the encoder. Inspired by Mamba, a gating mechanism derived from the decoder is introduced to dynamically adjust the activation weights of the fused output. This mechanism effectively suppresses irrelevant noise while fusing cross-scale information, thereby improving segmentation performance.
- Based on the two core modules described above, we propose MFDA-UNet, a segmentation network designed to balance computational efficiency and performance in medical imaging tasks. Experimental results on four benchmark datasets encompassing different modalities demonstrate that MFDA-UNet outperforms models based on other paradigms. This confirms the model’s capability to capture long-range feature dependencies and extract local detailed features.

## 2. Related Work

### 2.1. Transformer-Based Medical Image Segmentation Architectures

In the domain of medical image segmentation, Convolutional Neural Networks (CNNs)—such as the classic U-Net [1] and its variants—have long served as robust baselines. Convolution-based derivatives, including UNet++ [7], Residual U-Net [8], and DenseUNet [9], have demonstrated stable performance across various segmentation tasks. However, constrained by the localized receptive fields of convolutional kernels, these architectures face fundamental bottlenecks when modeling long-range spatial dependencies.

To overcome this limitation, Vision Transformers (ViTs) introduced the self-attention mechanism to image processing. Recognizing the complementary nature of attention mechanisms and convolutions, hybrid architectures such as TransUNet [10] have been proposed to integrate the local feature extraction capabilities of CNNs with the global dependency modeling of Transformers. However, the computational complexity of standard self-attention scales quadratically with the sequence length of high-resolution medical images, incurring prohibitive computational and memory overheads.

Subsequent architectures have proposed various strategies to address this challenge. For instance, representative models like the Swin Transformer [11] achieve linear complexity by computing attention within shifted local windows. Nevertheless, this restricted receptive field inherently sacrifices the capacity for pure global context modeling. Dilated attention [12] employs mixed dilation rates to simultaneously capture short- and long-range dependencies using a limited number of sampling points. Linear attention [13] methods circumvent the quadratic dependence on sequence length by approximating the standard softmax operation with alternative kernel functions. Despite this efficiency advantage, previous studies confirm that standard linear attention is often constrained by its limited expressive capacity.

### 2.2. Mamba-Based Medical Image Segmentation

Recently, Mamba [14]—an architecture based on SSMs [4]—has demonstrated massive potential in long-sequence modeling due to its selective scanning mechanism and linear complexity. VM-UNet [15] and Swin-UMamba [16] replaced the foundational convolutional blocks of the classic U-Net, drawing on the concept of Vision State Space (VSS). To balance information across different granularities, LKM-UNet [17] innovatively integrated a patching strategy, constructing dual pixel-level and patch-level state space models to achieve refined feature extraction from the local-micro to the global-macro level. In addition, VM-UNet-V2 [18] focused on addressing the pain points of multi-scale feature fusion; it not only refined skip connections using Convolutional Block Attention Modules (CBAMs) but also developed a dedicated Semantic and Detail Infusion (SDI) module, thereby significantly strengthening the deep interaction between low-level and high-level features.

Notably, Research by Han et al. [5] has revealed a mathematical equivalence between SSMs and linear attention, demonstrating that the core of Mamba’s efficiency fundamentally stems from its specific block structure and forget gate design. Inspired by this, MILA (Mamba-Inspired Linear Attention) [5] was proposed. Based on Mamba’s foundational block design, the traditional forget gate mechanism is replaced by three types of Positional Encodings. MLLA-UNet [19] employs MILA to construct its model and proposes a novel sampling strategy for multi-scale feature fusion. Similarly, MLAgg-UNet [20] introduces the MLAgg block, which integrates the Mamba architectural design with differential attention and develops a multi-scale fusion mechanism based on SS2D token scanning.

## 3. Method

**Figure 1:**
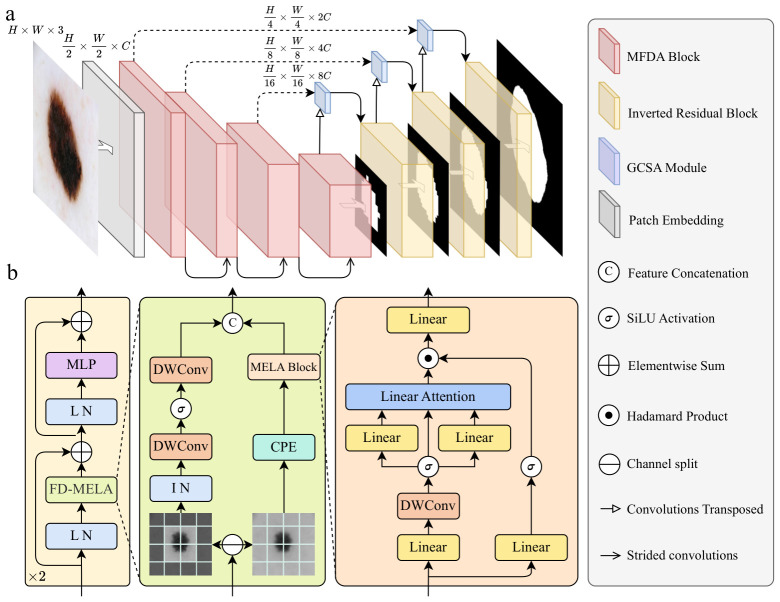
(**a**) The U-shaped encoder–decoder architecture of MFDA-UNet. (**b**) Structural schematic of the MFDA block.

The proposed MFDA-UNet framework, illustrated in Figure 1, adopts a U-shaped architecture that seamlessly integrates CNNs and Linear Attention. The core advantages lie in two key improvements: (1) MFDA Block: It adopts a hybrid architecture of convolution and linear attention. By introducing a frequency-decoupling mechanism, it leverages the respective strengths of both paradigms to independently process high-frequency local and low-frequency global features. This achieves a balance between the long-range modeling of large-scale organs and the perception of edge details. (2) Gated Cross-Scale Attention (GCSA) Module: This module aggregates multi-scale information via a cross-scale linear attention mechanism, efficiently fusing high-level semantic features from the decoder with structural detail features from the encoder. It utilizes the high-level semantics of the decoder to generate a dynamic gate, which adaptively re-weights the fused representations.

### 3.1. Overall Architecture

The proposed network adopts a standard U-shaped architecture. Drawing inspiration from [21], a patch embedding module is initially utilized to map the input image into a high-dimensional feature space. The encoder consists of four stages, where each stage uses two MFDA modules and a strided convolutional layer for downsampling to extract multi-scale features. During the feature recovery phase, the decoder combines inverted residual blocks and transposed convolutions to perform feature reconstruction and progressive upsampling. To achieve precise cross-scale feature fusion, a GCSA module is embedded at each hierarchical level. In addition, we introduce a deep supervision mechanism into the decoding path. By generating predicted masks at each scale to calculate auxiliary losses, this mechanism accelerates model convergence and improves segmentation accuracy.

### 3.2. Mamba-Inspired Frequency-Decoupled Attention

#### 3.2.1. Overall Architecture of MFDA

**Figure 2:**
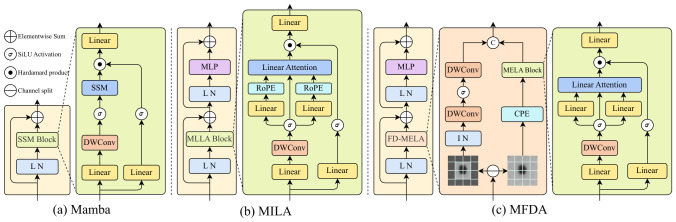
Structural comparison between the standard Mamba/MILA blocks and the proposed MFDA.

Figure 2 presents a direct structural comparison between our proposed MFDA and the Mamba and MILA blocks. The high efficiency of Mamba largely stems from its block structure and forget gate mechanism. MILA inherits this block structure and replaces the forget gate functionality with three types of Positional Encodings.

In contrast, while our proposed MFDA module substitutes the SSM with linear attention, it innovatively introduces a frequency decoupling mechanism, employing parallel branches to process high- and low-frequency data. To substitute the forget gate functionality, Conditional Positional Encoding (CPE) is incorporated into the low-frequency branch to supplement local features. Furthermore, in MILA’s MLLA block, Rotary Positional Embedding (RoPE) is applied to the queries (*Q*) and keys (*K*), while Local Enhanced Positional Encoding (LePE) is added to the values (*V*). Although MFDA similarly adopts LePE, it leverages frequency decoupling to dedicate the MELA block exclusively to mining low-frequency global information. This structural design naturally captures global contextual features, thereby eliminating the need to introduce RoPE. Consequently, this approach not only avoids functional duplication but also further improves computational efficiency.

This block follows the classic Transformer stacking paradigm, consisting of a feature mixer and a Multi-Layer Perceptron (MLP). Given an input feature $X\in R^{B\times C\times H\times W}$, it is first flattened into a 1D sequence $X_{seq}\in R^{B\times N\times C}$ and subjected to Layer Normalization (LN). Subsequently, the feature is fed into the core FD-MELA block. The output then undergoes DropPath regularization and is combined with a residual connection to obtain the intermediate feature $X^{\prime }$, formulated as

(1) $$X^{\prime }=X_{seq}+\mathrm{DropPath}\left(\mathrm{FD}\text{-}\mathrm{MELA}\left(\mathrm{LN}\left(X_{seq}\right)\right)\right)$$

Before entering the MLP, Conditional Positional Encoding (CPE) is introduced to $X^{\prime }$. This step utilizes a 3×3 depth-wise convolution (DWConv) to dynamically and implicitly inject local positional information:

(2) $$X^{\prime \prime }=X^{\prime }+\mathrm{DWConv}\left(X^{\prime }\right)$$

Finally, a second LN and the MLP are employed to complete the non-linear mapping in the channel dimension. Following another residual connection, the final output is defined as

(3) $$X_{out}=X^{\prime \prime }+\mathrm{DropPath}\left(\mathrm{MLP}\left(\mathrm{LN}\left(X^{\prime \prime }\right)\right)\right)$$

#### 3.2.2. FD-MELA Block

To independently model the diverse frequency components in medical images while maintaining strict linear computational complexity, the FD-MELA block equally divides the normalized features along the channel dimension into two parts: $X_{half1}\in R^{B\times N\times (C/2)}$ and $X_{half2}\in R^{B\times N\times (C/2)}$. Following recent advanced methods [22,23], we adopt an extremely simple average pooling approach to perform high–low frequency decomposition. Specifically, for the first half, the block extracts the low-frequency, spatially smooth components using a 3×3 average pooling operation, denoted as $X_{low}=\mathrm{AvgPool}\left(X_{half1}\right)$. For the second half, the high-frequency edge components are obtained by subtracting their smoothed features via a residual connection, as formulated in Equation (4):

(4) $$X_{high}=X_{half2}-\mathrm{AvgPool}\left(X_{half2}\right)$$

After separation, the high-frequency and low-frequency components enter independent branches for processing. The high-frequency branch is specifically designed to capture local fine-grained features: it first eliminates contrast shifts through InstanceNorm, then utilizes a DWConv with a 5×5 receptive field and a SiLU activation function for broad local topology extraction, and finally undergoes a 1×1 convolution for channel projection. By employing learnable depth-wise convolutions, the model can adaptively extract modality-specific high-frequency semantic features, such as pigmentation textures in dermoscopy images or anatomical boundaries in CT scans.

Within the low-frequency processing pathway, the MELA block (detailed in Section 3.2.3) is employed to extract global macroscopic semantics. After feature extraction in both branches, the processed high-frequency and low-frequency features are concatenated along the channel dimension and fused through a linear projection layer. This process fully leverages the respective strengths of convolutions and linear attention, achieving a deep decoupling and unified fusion of global semantics and local details with low computational overhead.

#### 3.2.3. MELA Block

Drawing on the findings in [5], Conditional Positional Encoding (CPE) is applied to the low-frequency components to substitute the forget gate functionality of Mamba, thereby supplementing input-dependent positional information. The calculation of CPE is formulated as Equation (2), yielding $X_{low}^{\prime }$.

Subsequently, the features $X_{low}^{\prime }$ are fed into the MELA block to establish long-range dependencies. The block adopts a parallel dual-branch design referring to the structure of gated linear units, aiming to filter redundant information through a learnable gating mechanism. In the gating branch, the feature $X_{low}^{\prime }\in R^{B\times N\times C}$ is processed by a linear layer and a SiLU activation function to generate the gating signal $G\in R^{B\times N\times C}$. The main processing branch handles core feature extraction. Here, $X_{low}^{\prime }$ first undergoes linear projection and is reshaped to $R^{B\times C\times H\times W}$. It then aggregates local topological features via a 2D depth-wise separable convolution. After passing through the SiLU activation function, the dimensions are flattened back to $R^{B\times N\times C}$. The preliminary calculation process of the two branches is shown in Equation (5)

(5) $$Z=\sigma \left(\mathrm{DWConv}\left(\mathrm{Linear}\left(X_{low}^{\prime }\right)\right)\right),\;G=\sigma \left(\mathrm{Linear}\left(X_{low}^{\prime }\right)\right)$$

Here, σ represents the SiLU activation function. The output of the main branch *Z* subsequently enters the linear attention core module. First, linear layers map the feature *Z* respectively, and an exponential linear unit (ELU) is utilized to ensure feature non-negativity, yielding the query matrix *Q* and key matrix *K*. The value matrix *V* is set strictly equal to *Z* to minimize the loss of original information. The calculation is shown in Equation (6):

(6) $$Q=\phi \left(ZW_{Q}\right),\;K=\phi \left(ZW_{K}\right),\;V=Z$$

Here, ϕ(·) denotes the kernel mapping function ELU(·)+1, while $W_{Q}$ and $W_{K}$ are learnable weight matrices.

Traditional self-attention mechanisms suffer from a computational complexity of $O(N^{2})$. MELA utilizes the associative property of matrix multiplication to prioritize the computation of $K^{⊤}V$, successfully reducing the complexity to O(N), which is linear with respect to the sequence length. Incorporating the scaling operation that divides both *K* and *V* by $\sqrt{N}$, the feature output *Y* of the linear attention is calculated as shown in Equation (7):

(7) $$Y=Q\left(\frac{1}{N}K^{⊤}V\right)⊙\rho$$

Here, ⊙ represents the Hadamard product, and ρ is a normalization factor used to maintain the numerical balance of the attention weights. Based on the implementation logic, ρ is obtained by the inner product of *Q* and the mean vector of *K* along the sequence dimension, calculated as shown in Equation (8):

(8) $$\rho =\frac{1}{Q{\left(\frac{1}{N}\sum_{j=1}^{N}K_{j}\right)}^{⊤}+\epsilon }$$

ϵ is a tiny constant preventing zero-division errors, which is set to $1\times 10^{-6}$ in this paper. To compensate for the deficiency of pure linear attention in local feature perception, the model introduces a local enhanced Positional Encoding (LePE) to *V*. After reshaping *V* into a 2D spatial structure, a 3×3 2D depth-wise convolution directly extracts neighborhood features and supplements them into the linear attention output in a residual format. The calculation of LePE is shown in Equation (9):

(9) LePE(V)=DWConv(V)

Finally, the linear attention output compensated with LePE is fused with the signal *G* generated by the gating branch using the Hadamard product, achieving adaptive feature activation. The fused feature then undergoes a linear projection layer and DropPath processing to obtain the final output of the block.

### 3.3. Gated Cross-Scale Attention Module

**Figure 3:**
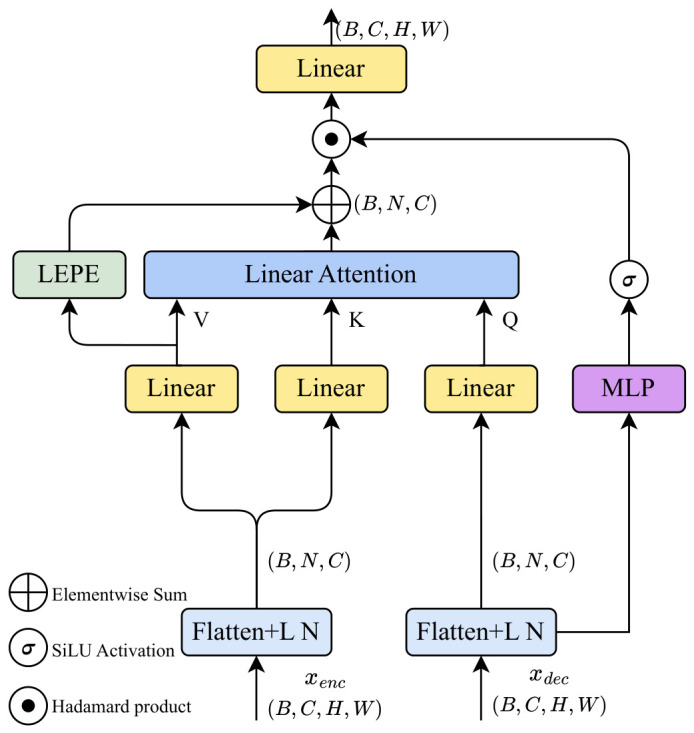
Architecture of the GCSA module.

Inspired by the gating mechanism in the Mamba block [14], we propose the Gated Cross-Scale Attention (GCSA) module. GCSA aggregates features via cross-scale linear attention and leverages deep semantic cues from the decoder to adaptively gate the globally fused representations, as illustrated in Figure 3.

The shallow encoder feature $X_{\mathrm{enc}}\in R^{B\times C\times H\times W}$ and the deep upsampled decoder feature $X_{\mathrm{dec}}\in R^{B\times C\times H\times W}$ are first flattened into 1D sequences and processed by LayerNorm. To establish cross-scale information interaction, the GCSA module uses linear layers to map the deep decoder feature into the query vector $Q\in R^{B\times N\times C}$ and maps the shallow encoder feature into the key vector $K\in R^{B\times N\times C}$ and the value vector $V\in R^{B\times N\times C}$, where N=H×W denotes the spatial sequence length.

Following the linear attention mechanism previously described in Section 3.2.3, *Q* and *K* are activated by the kernel function ϕ(·), and the cross-scale attention output is efficiently computed with O(N) complexity according to Equations (7) and (8). Subsequently, to compensate for local spatial details, the LEPE is applied to *V*, as defined in Equation (9), and is added to the linear attention output via a residual connection to obtain the intermediate fused feature *Y*.

Drawing on the findings in [5], Mamba’s gating mechanism is a key factor driving its performance improvements. Therefore, this module introduces an adaptive gating mechanism to dynamically filter the aggregated representations. Given that the decoder contains rich high-level semantics, the deep feature $X_{\mathrm{dec}}$ undergoes a non-linear transformation through an MLP, composed of two linear projection layers and a GELU activation. The output is then scaled by a Sigmoid function to generate the gating weight matrix *G*. Finally, $G\in R^{B\times N\times C}$ is multiplied with the intermediate fused feature $Y\in R^{B\times N\times C}$ via the Hadamard product, followed by a final linear projection layer to restore the channel dimension, yielding the final output $Y^{\prime }$. The complete gating and output calculation can be formulated as Equation (10):

(10) $$G=\sigma \left(\mathrm{MLP}\left(X_{\mathrm{dec}}\right)\right),\;Y^{\prime }=\mathrm{Linear}\left(Y⊙G\right)$$

where σ denotes the Sigmoid function and ⊙ represents the Hadamard product. Subsequently, the 1D sequence $Y^{\prime }$ is reshaped back to the 2D spatial format $R^{B\times C\times H\times W}$ to ensure structural compatibility for subsequent feature fusion. By utilizing the high-level semantic cues from the decoder to selectively gate the cross-scale aggregated features, the GCSA module effectively eliminates noise interference from shallow layers and accurately bridges the semantic gap between the encoder and decoder.

## 4. Experiments

### 4.1. Datasets

- ISIC 2017 and ISIC 2018 Datasets [24,25]: The International Skin Imaging Collaboration 2017 and 2018 challenge datasets are two publicly available skin lesion segmentation datasets, containing 2150 and 2694 dermoscopy images with segmentation mask labels, respectively. Following previous works [15,26], we split the datasets in a 7:3 ratio for use as training and test sets. Specifically, for the ISIC17 dataset, the training set consists of 1500 images, and the test set consists of 650 images. For the ISIC18 dataset, the training set includes 1886 images, while the test set contains 808 images. For these two datasets, we provide detailed evaluations on several metrics, including Mean Intersection over Union (mIoU), Dice Similarity Coefficient (DSC), Accuracy (Acc), Sensitivity (Sen), and Specificity (Spe).
- Synapse Dataset [27]: The publicly accessible Synapse dataset contains 30 clinical abdominal CT scans, yielding a total of 3779 axial images. Its primary objective is the multi-class segmentation of eight abdominal organs. Following previous works [10,15], we allocate 18 cases to the training set and the remaining 12 to the test set. Segmentation accuracy is quantified using two standard metrics: the 95% Hausdorff Distance (HD95) and the Dice Similarity Coefficient (DSC).
- ACDC Dataset [28]: The Automated Cardiac Diagnosis Challenge dataset provides cardiac MRI scans from 100 patients. The segmentation targets include three main regions: the myocardium, right ventricle, and left ventricle. Following previous studies [10,29], we partitioned the full dataset into 70, 10, and 20 cases for training, validation, and testing, respectively. We adopted the Dice Similarity Coefficient (DSC) as the primary metric for quantitative performance assessment.

### 4.2. Implementation Details

Following prior works [15,29], to maintain consistency with standard evaluation procedures, all samples from the ISIC datasets are resized to a spatial resolution of 256×256, while images from the Synapse and ACDC benchmarks are adjusted to 224×224. Dynamic spatial augmentations, including random flipping and rotation, are applied during training to prevent overfitting. For the optimization objective, a hybrid BCE-Dice loss is utilized for the binary skin lesion segmentation tasks, whereas a combined CE-Dice loss is employed for the multi-class anatomical tasks.

The network parameters are updated using the AdamW [30] optimizer with an initial learning rate of $1\times 10^{-3}$. During training, the batch size is set to 24 for the ISIC datasets and 32 for the Synapse and ACDC benchmarks. To ensure smooth convergence, the learning rate is progressively decayed to a minimum of $1\times 10^{-5}$ using a cosine annealing scheduler throughout the total training epochs. To demonstrate its inherent representational capability, our model is trained entirely from scratch without pre-trained weights, with all experiments conducted on a single NVIDIA RTX 4090 GPU (NVIDIA Corporation, Santa Clara, CA, USA).

To ensure a standardized and fair evaluation, we followed the data processing protocols detailed in Section 4.1. Furthermore, the baseline results for the ISIC 2017, ISIC 2018, and Synapse datasets are directly cited from [15], while the baseline results for the ACDC dataset are cited from [29].

### 4.3. Quantitative and Qualitative Segmentation Results

#### 4.3.1. ISIC 2017 and ISIC 2018 Datasets

Results for the ISIC 2017 dataset are summarized in Table 1. MFDA-UNet achieves the best performance in four of five evaluation metrics, with an mIoU of 80.94% and a DSC of 89.47%. Compared to the non-pre-trained VM-UNet*, the mIoU and DSC improve by 3.35% and 2.09%, respectively; even compared to the pre-trained VM-UNet, the mIoU of MFDA-UNet still improves by 0.71% and the DSC improves by 0.44%.

A key advantage of MFDA-UNet lies in the equilibrium between Specificity and Sensitivity. In comparison to MALUNet, which secures a high Specificity of 98.47% at the cost of a significant drop in Sensitivity (84.78%), MFDA-UNet attains a higher Specificity of 98.48% while maintaining a robust Sensitivity of 87.02%.

Results for the ISIC 2018 dataset are summarized in Table 2. MFDA-UNet achieves an mIoU of 81.59% and a DSC of 89.86%. Compared to the non-pre-trained VM-UNet, the mIoU and DSC improve by 2.93% and 1.8%, respectively. When evaluated against the heavily pre-trained VM-UNet, the performance gains appear relatively marginal, yielding increases of only 0.24% in mIoU and 0.15% in DSC.

To rigorously verify that the performance improvement of MFDA-UNet stems from its intrinsic architectural advantages, we conducted a repeated-run statistical analysis on the ISIC 2018 dataset. Over three independent runs with different random seeds, MFDA-UNet achieved an average DSC of 89.83 ± 0.03% (95% CI: [89.75%, 89.91%]) and an average mIoU of 81.55 ± 0.04%. A one-sample *t*-test comparing our multi-run DSC distribution against the pre-trained VM-UNet baseline (89.71%) yielded a *p*-value < 0.05. This statistical result confirms that the performance superiority of MFDA-UNet is highly robust.

**Figure 4:**
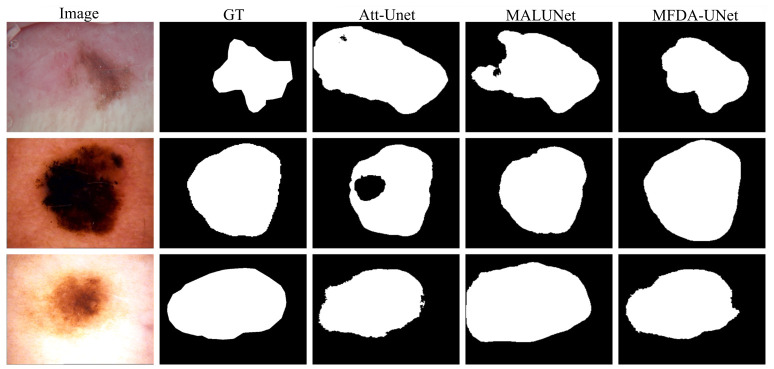
The segmentation results of different methods on the ISIC2018 dataset.

Figure 4 presents a visual comparison on ISIC2018. As observed from the results, Att-Unet exhibits obvious performance deficiencies. In the lesion sample in the second row, Att-Unet suffers from severe internal under-segmentation, resulting in large segmentation voids in the core region and failing to depict the overall structure of the lesion. Furthermore, in the low-contrast sample in the first row, this model demonstrates weak perceptual ability for fine-grained edges. Its predicted contours are accompanied by numerous jagged artifacts and irregular protrusions.

As seen in the first row, MALUNet falls short in precise boundary delineation, leading to abnormal protrusions and indentations that deviate from the true contour. In contrast, the proposed MFDA-UNet achieves the optimal segmentation performance across all test samples. Benefiting from frequency decoupling, MFDA-UNet not only macroscopically localizes the lesion but also captures irregular contours and soft boundary transitions, producing smooth segmentation edges without obvious artifacts.

#### 4.3.2. Synapse Dataset

Table 3 presents the quantitative results of MFDA-UNet compared with several advanced networks on the Synapse multi-organ dataset.

MFDA-UNet achieves the highest overall performance with a mean DSC of 81.61%. Additionally, its HD95 of 19.97 mm remains within the leading tier, but is slightly inferior to MEW-Unet and VM-Unet. This indicates that there is still room for further optimization in boundary segmentation. In terms of specific organs, MFDA-UNet exhibits exceptional feature extraction capabilities for complex anatomical targets. Most notably, on the notoriously difficult gallbladder—a small organ with high inter-subject variability—MFDA-UNet achieves a DSC of 73.37%, marking a significant leap of nearly 4% over the VM-UNet. This success is primarily attributed to the MFDA and GCSA modules, which effectively bridge deep semantics and fine-grained spatial details to prevent performance imbalances between easy and challenging organs.

**Figure 5:**
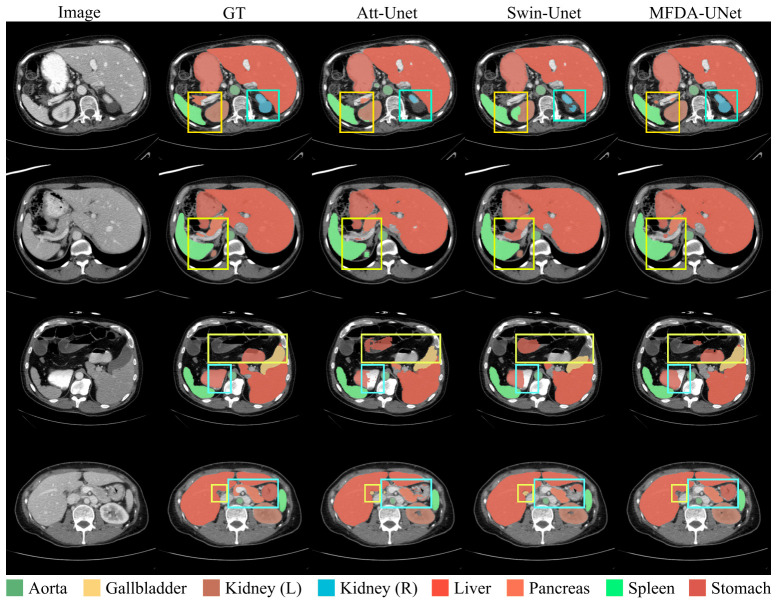
The segmentation results of different methods on the Synapse multi-organ CT dataset.

Figure 5 illustrates the 2D slice comparisons. For example, in the boxed regions of the first and third rows, organs such as the kidneys, stomach, and pancreas exhibit low contrast with surrounding tissues and possess blurred boundaries. Att-Unet and Swin-Unet exhibit pronounced errors in these areas. They suffer from severe under-segmentation of the target organs, producing internal holes, while simultaneously generating numerous false positive predictions in the surrounding background regions. Conversely, within the cyan boxed region of the fourth row, MFDA-UNet successfully and completely delineates the entire structure of the stomach, whereas the other two models exhibit substantial segmentation errors.

**Figure 6:**
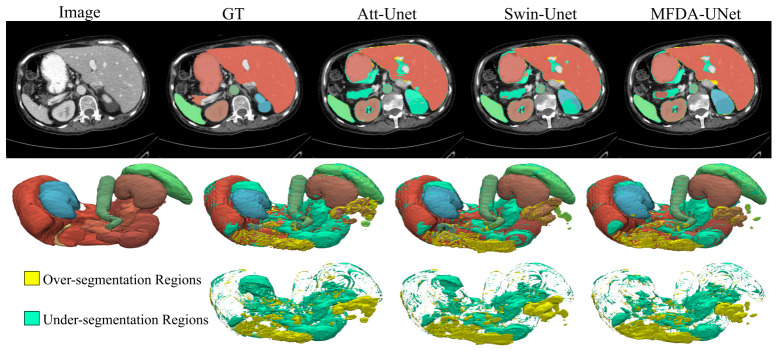
The segmentation results of different methods on the Synapse multi-organ CT dataset. The first row displays the 2D CT slices and their corresponding segmentation results. The second row shows the 3D visualization of the reconstructed organs, while the third row illustrates the 3D visualization of the error regions.The bright yellow areas indicate over-segmentation regions, and the cyan areas represent under-segmentation regions.

As can be seen from the 2D slice results in the first row of Figure 6, the organ morphology generated by Att-Unet exhibits obvious topological errors and surface burrs. Although Swin-Unet shows some improvement, the boundaries of complex structures remain unsmooth. Both models suffer from varying degrees of missed detection in the blue-labeled kidney region. In contrast, the proposed MFDA-UNet achieves complete segmentation of the kidney. Regarding pancreas segmentation, all three models exhibit notable missed detections; MFDA-UNet retains more correctly segmented areas.

The second and third rows of Figure 6 present the 3D organ reconstruction results and 3D error distribution maps, respectively. It can be clearly observed that the error maps of Att-Unet and Swin-Unet contain massive, contiguous patches of yellow and cyan error regions, whereas our model produces the fewest and most scattered error regions. Specifically, according to the error visualization in the third row, Att-Unet suffers from large areas of under-segmentation in the blue-labeled kidney region located in the upper left corner. In contrast, MFDA-UNet correctly captures the overall tissue structure and mass of the kidney. These visual results intuitively demonstrate MFDA-UNet’s robust segmentation capability. The model effectively suppresses background noise and substantially decreases major topological errors.

#### 4.3.3. ACDC Dataset

As highlighted by nnU-Net [41], the ACDC dataset is a highly effective benchmark for validating model robustness. We conducted further experiments on this dataset, with quantitative results presented in Table 4. The proposed MFDA-UNet achieves the highest average DSC of 90.84%, outperforming the pre-trained SwinUnet by 0.84%. Notably, for the highly challenging myocardium (Myo), MFDA-UNet achieves a 1.30% improvement over SwinUnet. These results demonstrate that the synergy of frequency decoupling and cross-scale linear attention enables robust feature representation and accurate localization of complex anatomical structures, confirming MFDA-UNet’s exceptional overall performance.

### 4.4. Ablation Study

To evaluate the individual contributions and synergistic effects of the core components in MFDA-UNet, we conduct ablation studies on the ISIC 2018 dataset. As shown in Table 5, the pure convolutional baseline (first row), utilizing standard skip connections, achieves a DSC of 87.79% with 13.73 M parameters and 19.82 G FLOPs.

We first verify the necessity of the frequency-decoupled mechanism within the MFDA block. When employing a version without frequency decoupling (w/o FD) in the second row, the DSC improves to 88.54%, but the parameters surge to 26.06 M. This is because performing linear attention calculations across all feature channels incurs massive parameter overhead. Furthermore, we evaluate the variant without Positional Encoding (w/o PE) in the third row, which achieves a DSC of 88.71%. In contrast, incorporating the complete MFDA block (fourth row) only slightly increases the parameters to 16.97 M but effectively improves the DSC to 89.19%. These results demonstrate that frequency decoupling can effectively eliminate parameter redundancy while improving performance and simultaneously introducing Positional Encoding to substitute the forget gate functionality can further enhance segmentation accuracy.

Next, by replacing standard skip connections with the GCSA module (fifth row), the model achieves a DSC of 88.87% with a parameter increase of only 0.78 M. This confirms that GCSA can better fuse the features of the encoder and decoder.

Furthermore, we evaluate the impact of the deep supervision strategy. As seen in the sixth row, removing deep supervision (w/o DS) from the combined model results in a slight performance drop, yielding a DSC of 89.59%.

Finally, the complete MFDA-UNet model (seventh row) achieves a peak DSC of 89.86%, yielding a 2.07% absolute improvement over the baseline, with its parameters and FLOPs increasing by only 4.42 M and 4.79 G, respectively. These results validate the synergy of all the aforementioned components, providing an optimized solution for high-performance medical image segmentation.

To investigate integrating Mamba’s gating design, we evaluate four configurations (Table 6). Compared to the convolutional baseline (Index a), introducing the ungated GCSA (Index b) noticeably improves mIoU to 79.36% and DSC to 88.49%. We then compared two gating positions. Applying the gate to the encoder’s value projection (*V*) (Index c) yields only limited improvements. In contrast, applying the dynamic gate to the final aggregated output (Index d) achieves the best performance, peaking at 79.97% mIoU and 88.87% DSC. This confirms that adaptively filtering fused features—guided by decoder semantics—is the optimal way to adapt Mamba’s principles in U-shaped networks.

## 5. Conclusions

To address the challenges of blurred boundaries, variable lesion scales, and complex textures in medical images, this paper proposes MFDA-UNet, a U-shaped segmentation architecture based on frequency decoupling and cross-scale linear attention. In the encoder, we introduce the Mamba-inspired Frequency-Decoupled Attention (MFDA) block, which explicitly decomposes features: the low-frequency branch captures global dependencies via linear-complexity attention, while the high-frequency branch preserves local edge details using Depthwise Separable convolutions. Furthermore, to bridge the semantic gap during decoding, a novel GCSA module is integrated into the skip connections. This module utilizes a cross-scale linear attention mechanism to achieve cross-scale information fusion and leverages the decoder’s deep semantic features to compute an adaptive gate for feature filtering.

We conducted comprehensive experiments on four public benchmarks: ISIC 2017, ISIC 2018, Synapse, and ACDC. MFDA-UNet achieved DSC scores of 89.47%, 89.86%, 81.61%, and 90.84% across the respective datasets, outperforming existing mainstream models.

Finally, step-wise ablation studies systematically validate the efficacy of our proposed components. Experimental results demonstrate that the frequency decoupling mechanism enhances representation capability while substantially reducing parameter redundancy, and Positional Encoding can further improve segmentation performance. The GCSA module is vital for fusing cross-scale spatial details. Furthermore, our exploration of the gating adaptation reveals that applying the gate to filter the fused results achieves optimal performance. Additionally, the inclusion of deep supervision further stabilizes the training process and boosts the final segmentation accuracy. In summary, adopting a hybrid architecture of convolutions and linear transformers, MFDA-UNet achieves an a highly competitive balance between global context modeling and local detail recovery via frequency decoupling. By leveraging cross-scale linear attention and the gating mechanism for information fusion and selection, it provides a highly efficient and robust solution for complex medical image segmentation tasks.

## Figures and Tables

**Table 1:** Quantitative results on the ISIC 2017 dataset, formatted as a heatmap where green, yellow, and orange cells correspond to the best, intermediate, and relatively poor performances. VM-UNet* denotes the model version trained from scratch.

Method	ISIC 2017				
	mIoU (%)	DSC (%)	Acc (%)	Spe (%)	Sen (%)
Unet [1]	76.98	86.99	95.65	97.43	86.82
UTNetV2 [31]	77.35	87.23	95.84	98.05	84.85
TransFuse [32]	79.21	88.40	96.17	97.98	87.14
MALUNet [26]	78.78	88.13	96.18	98.47	84.78
VM-Unet* [15]	77.59	87.38	-	-	-
VM-UNet [15]	80.23	89.03	96.29	97.58	89.90
MFDA-UNet	80.94	89.47	96.57	98.48	87.02

**Table 2:** Quantitative results on the ISIC 2018 dataset, formatted as a heatmap where green, yellow, and orange cells correspond to the best, intermediate, and relatively poor performances. VM-UNet* denotes the model version trained from scratch.

Method	ISIC 2018				
	mIoU (%)	DSC (%)	Acc (%)	Spe (%)	Sen (%)
UNet [1]	77.86	87.55	94.05	96.69	85.86
UNet++ [7]	78.31	87.83	94.02	95.75	88.65
Att-Unet [33]	78.43	87.91	94.13	96.23	87.60
UTNetV2 [31]	78.97	88.25	94.32	96.48	87.60
SANet [34]	79.52	88.59	94.39	95.97	89.46
TransFuse [32]	80.63	89.27	94.66	95.74	91.28
MALUNet [26]	80.25	89.04	94.62	96.19	89.74
VM-Unet* [15]	78.66	88.06	-	-	-
VM-Unet [15]	81.35	89.71	94.91	96.13	91.12
MFDA-UNet	81.59	89.86	95.21	97.70	87.50

**Table 3:** Quantitative results on the Synapse dataset, formatted as a heatmap where green, yellow, and orange cells correspond to the best, intermediate, and relatively poor performances. The evaluation metric for all individual organs is the DSC.

Methods	DSC	HD95	Aorta	Gallbladder	Kidney (L)	Kidney (R)	Liver	Pancreas	Spleen	Stomach
V-Net [35]	68.81	-	75.34	51.87	77.10	80.75	87.84	40.05	80.56	56.98
DARR [36]	69.77	-	74.74	53.77	72.31	73.24	94.08	54.18	89.90	45.96
R50 U-Net [10]	74.68	36.87	87.47	66.36	80.60	78.19	93.74	56.90	85.87	74.16
Unet [1]	76.85	39.70	89.07	69.72	77.77	68.60	93.43	53.98	86.67	75.58
R50 Att-Unet [10]	75.57	36.97	55.92	63.91	79.20	72.71	93.56	49.37	87.19	74.95
Att-Unet [10]	77.77	36.02	89.55	68.88	77.98	71.11	93.57	58.04	87.30	75.75
R50 ViT [10]	71.29	32.87	73.73	55.13	75.80	72.20	91.51	45.99	81.99	73.95
TransUNet [10]	77.48	31.69	87.23	63.13	81.87	77.02	94.08	55.86	85.08	75.62
TransNorm [37]	78.40	30.25	86.23	65.10	82.18	78.63	94.22	55.34	89.50	76.01
Swin-Unet [29]	79.13	21.55	85.47	66.53	83.28	79.61	94.29	56.58	90.66	76.60
TransDeepLab [38]	80.16	21.25	86.04	69.16	84.08	79.88	93.53	61.19	89.00	78.40
MT-Unet [39]	78.59	26.59	87.92	64.99	81.47	77.29	93.06	59.46	87.75	76.81
MEW-Unet [40]	78.92	16.44	86.68	65.32	82.87	80.02	93.63	58.36	90.19	74.26
VM-Unet [15]	81.08	19.21	86.40	69.41	86.16	82.76	94.17	58.80	89.51	81.40
MFDA-UNet	81.61	19.97	88.16	73.37	85.00	81.58	95.10	58.13	90.80	80.77

**Table 4:** Quantitative results on the ACDC dataset, formatted as a heatmap where green, yellow, and orange cells correspond to the best, intermediate, and relatively poor performances. The evaluation metric for all individual organs is the DSC.

Method	ACDC			
	DSC (%)	RV	Myo	LV
R50 U-Net [10]	87.55	87.10	80.63	94.92
R50 Att-Unet [10]	86.75	87.58	79.20	93.47
R50 ViT [10]	87.57	86.07	81.88	94.75
TransUnet [10]	89.71	88.86	84.53	95.73
Swin-Unet [29]	90.00	88.55	85.62	95.83
MFDA-UNet	90.84	89.45	86.92	96.15

**Table 5:** Ablation study of different components on the ISIC2018 dataset, formatted as a heatmap where green, yellow, and orange cells correspond to the best, intermediate, and relatively poor performances. The abbreviation “w/o FD” denotes the model variant without the frequency decoupling mechanism, “w/o DS” indicates the variant without deep supervision, and “w/o PE” represents the variant without Positional Encoding.

	MFDA	GCSA	w/o FD	w/o PE	w/o DS	DSC	Params (M)	FLOPs (G)
**Methods**						87.79	13.73	19.82
	✓		✓			88.54	26.06	21.67
	✓			✓		88.71	16.90	21.75
	✓					89.19	16.97	21.84
		✓				88.87	14.51	21.68
	✓	✓			✓	89.59	18.15	24.61
	✓	✓				89.86	18.15	24.61

**Table 6:** Comparison of different adaptation strategies for the gating mechanism in the GCSA module.

Index	mIoU (%)	DSC (%)	Acc (%)	Spe (%)	Sen (%)
a	78.24	87.79	91.22	92.05	87.62
b	79.36	88.49	91.76	91.58	89.24
c	79.57	88.62	91.83	93.12	87.88
d	79.97	88.87	92.08	93.26	88.95

## Data Availability

The original contributions presented in the study are included in the article. Further inquiries can be directed to the corresponding author.
